# Obstetricians’ Opinions of the Optimal Caesarean Rate: A Global Survey

**DOI:** 10.1371/journal.pone.0152779

**Published:** 2016-03-31

**Authors:** Francesca L. Cavallaro, Jenny A. Cresswell, Carine Ronsmans

**Affiliations:** Department of Infectious Disease Epidemiology, London School of Hygiene & Tropical Medicine, London, United Kingdom; Oslo University Hospital, Ullevål, NORWAY

## Abstract

**Background:**

The debate surrounding the optimal caesarean rate has been ongoing for several decades, with the WHO recommending an “acceptable” rate of 5–15% since 1997, despite a weak evidence base. Global expert opinion from obstetric care providers on the optimal caesarean rate has not been documented. The objective of this study was to examine providers’ opinions of the optimal caesarean rate worldwide, among all deliveries and within specific sub-groups of deliveries.

**Methods:**

A global online survey of medical doctors who had performed at least one caesarean in the last five years was conducted between August 2013 and January 2014. Respondents were asked to report their opinion of the optimal caesarean rate—defined as the caesarean rate that would minimise poor maternal and perinatal outcomes—at the population level and within specific sub-groups of deliveries (including women with demographic and clinical risk factors for caesareans). Median reported optimal rates and corresponding inter-quartile ranges (IQRs) were calculated for the sample, and stratified according to national caesarean rate, institutional caesarean rate, facility level, and respondent characteristics.

**Results:**

Responses were collected from 1,057 medical doctors from 96 countries. The median reported optimal caesarean rate was 20% (IQR: 15–30%) for all deliveries. Providers in private for-profit facilities and in facilities with high institutional rates reported optimal rates of 30% or above, while those in Europe, in public facilities and in facilities with low institutional rates reported rates of 15% or less. Reported optimal rates were lowest among low-risk deliveries and highest for Absolute Maternal Indications (AMIs), with wide IQRs observed for most categories other than AMIs.

**Conclusions:**

Three-quarters of respondents reported an optimal caesarean rate above the WHO 15% upper threshold. There was substantial variation in responses, highlighting a lack of consensus around which women are in need of a caesarean among obstetric care providers worldwide.

## Introduction

Large differences exist between national caesarean rates worldwide, from 1.4% of all deliveries in Niger in 2012 to 52.3% in Brazil in 2010 [1], reflecting both differences in access to caesareans as well as in delivery care practices. The debate around the “optimal” caesarean rate is ongoing: the WHO has recommended since 1997 that population-based caesarean rates should be between 5–15% [2], which was upheld in the 2009 revised guidelines for monitoring emergency obstetric care [3]. A number of studies published after these guidelines [4–12] (summarised in a systematic review [13]) investigated the ecological relationship between national caesarean rates and mortality, and showed that maternal and neonatal mortality decline as the caesarean rate rises, up until a certain threshold. The thresholds reported by these studies range between 9–16% for maternal and infant or neonatal mortality, although most studies did not control for confounders. In their recent statement [14], the WHO suggested that population-level rates above 10% are not justified, based on the finding in their own ecological study of no association between caesarean rates and maternal or neonatal mortality above this threshold, after adjusting for socioeconomic development [15]. However, another recent ecological study reported that maternal and neonatal mortality continue to decline with caesarean rates up to 19%, after adjusting for several national indicators [16].

Importantly, these ecological studies do not take into account morbidity of the mother or baby, and no optimal caesarean rate minimising both mortality and morbidity has been identified. In the absence of an evidence-based optimal caesarean rate for maternal and perinatal outcomes, expert opinion may be helpful to assess to what extent obstetricians agree with the WHO recommendation. Obstetricians’ opinions have been recognised as an important driver of caesarean section rates [17, 18], and documenting variation in opinions of the optimal rate is useful for understanding differences in medical childbirth environments. One survey of South African obstetricians conducted in 1992 found that the “ideal” rate was considered to be 20% among private providers and 16% among public providers [19], though the authors did not explore agreement between respondents. The wide variation in caesarean rates across countries and between health facilities suggests that opinions of the optimal caesarean rate would increase with national and institutional caesarean rates, and be higher among doctors practicing in private compared to public facilities.

Furthermore, the question of optimal rates among specific sub-groups of deliveries is unresolved. While the Robson classification has become increasingly used to analyse patterns of “over-intervention” within health facilities, there is no agreement on what thresholds indicate unnecessary caesareans. Although there is likely to be little disagreement that most Absolute Maternal Indications (AMIs), such as major cephalo-pelvic disproportion and complete placenta praevia, require surgery to avert the death of the mother [20], for other conditions (such as twin deliveries and women with previous caesareans) caesarean sections may sometimes—but not always—be necessary. The evidence from randomised controlled trials is either lacking or inconclusive [21, 22], potentially leaving room for wide variations in opinions and practices. Examining obstetricians’ opinions of the optimal caesarean rate among specific categories of deliveries would help assess the relative magnitude of perceived need for caesareans in different groups of women.

Previous studies have documented differences in obstetricians’ attitudes towards caesareans on maternal request [23–28] and personal preferences for mode of delivery for uncomplicated births [24, 26, 28–30] in high-income settings. To our knowledge, this is the first study to examine global variation in obstetricians’ opinions of the optimal caesarean rate. The objective of our study was to determine the extent of variation in reported optimal rates for all deliveries and within specific categories of deliveries, and to evaluate whether reported optimal caesarean rates vary according to national caesarean rate, institutional caesarean rate, facility type, facility level and respondent characteristics.

## Methods

### Study design and population

A cross-sectional online survey was designed to collect doctors’ opinions of the optimal caesarean rate. In this study, the optimal caesarean rate was defined as the “proportion of women who should receive a caesarean for optimal maternal and fetal outcomes”, including mortality and morbidity. The target population were medical doctors having performed at least one caesarean in the last five years, worldwide (collectively referred to as “obstetricians” throughout this paper).

### Questionnaire development

A questionnaire was developed to collect respondent characteristics (age, gender, occupation, main country of practice, as well as the type (public, private for-profit and private not-for-profit), level and caesarean rate of the facility where they most recently provided obstetric care). Reported optimal caesarean rate for all deliveries was collected in free text format, whilst reported optimal rates among specified categories of deliveries was collected in 10% intervals (0%, 1–10%, 11–20% […] 91–100%). The wording of the question for reporting optimal caesarean rates was adapted from an unpublished survey asking experts for their opinion on “plausible” rates of emergency caesareans [31]. The questionnaire was piloted in June and July 2013 to assess face validity and refine the wording of the question. The final question was worded as “Of 1000 women with the following characteristic [_______], what proportion should receive a caesarean for optimal maternal and fetal outcomes?”

Respondents were also asked to report the optimal caesarean rate for four groups of deliveries, which were organised from high to low hypothesised need for caesareans. “Absolute Maternal Indications” (AMIs) are complications for which surgery is thought necessary to avert the death of the mother [20], and which require a caesarean in all cases. “Other clinical categories” include clinical conditions for which there is thought to be an elevated need for caesareans, but not all women with these complications require a caesarean (such as women with prolonged labour). “Reproductive categories” include more distal risk factors for obstetric complications which may be associated with higher need for caesarean: for example, low maternal height is thought to be a risk factor for small pelvic size, and thus for major cephalopelvic disproportion [32]. The lowest risk category of deliveries is considered to be multipara with a singleton cephalic delivery, no previous caesarean, and no risk factors known at the onset of delivery.

### Participant recruitment

The final version of the questionnaire was translated into French, Portuguese and Spanish, and translated versions were checked and edited by native speakers with medical training. The survey was uploaded via the online platform SurveyMonkey (https://www.surveymonkey.com), and was accessible from 14^th^ August 2013 to 31^st^ January 2014. Respondents gave informed consent by checking a box stating they had understood the information and terms of participation before proceeding to the survey, and respondents were able to exit the survey at any stage. Ethical approval for this study was granted by the Research Ethics Committee of the London School of Hygiene & Tropical Medicine (no 6455).

It was not possible to achieve a probability sample since there is no sampling frame for this population and the response rate is unknown: an unknown proportion of obstetricians is unreachable online, and surveys of medical doctors in high-income countries tend to have low response rates [23, 24, 29, 30]. A multi-pronged approach to distribution was adopted with the aim of recruiting the largest and most geographically diverse sample possible, by disseminating the survey via 32 national obstetrics societies to their members (Bolivia, Burkina Faso, Denmark, Ecuador, Ghana, Haiti, Honduras, Iceland, Kenya, Lebanon, Luxemburg, Malawi, Malaysia, Mexico, Mozambique, Nepal, Nigeria, Norway, Papua New Guinea, Rwanda, Singapore, Slovenia, South Africa, Spain, Sudan, Switzerland, Taiwan, Thailand, Turkey, Uganda, and United Kingdom). Other maternal health organisations also distributed the survey among their networks, and collaborators of large multi-centre studies in obstetric care (WHO Global Survey on Maternal and Perinatal Health, WHO INTERGROWTH-21^st^, FEMHealth, and the WOMAN Trial) were invited to participate. The social networking sites Facebook and Twitter were also used, and snowball sampling was encouraged by enabling respondents to share the survey with their colleagues.

### Explanatory variables and sources

The main explanatory variable of interest in this study was the national caesarean rate in the respondents’ main country of practice, grouped into four categories (<5%, 5–14.9%, 15–29.9%, ≥30%). The list of national caesarean rates compiled by Gibbons et al. in 2012 [33] was updated with the most recent available estimates at the time of the survey from the WHO Global Health Observatory [1] and Demographic and Health Survey reports [34] (S1 Table). Geographical region was categorised according to the WHO classification [35], and country income level according to the World Bank classification [36].

### Statistical analyses

The median reported optimal caesarean rate was calculated for all deliveries along with the corresponding interquartile range (IQR). Where respondents responded by providing an optimal range (e.g. 15–20%), the interval midpoint was used to calculate the median optimal caesarean rate for the sample (e.g. 17.5%). For clinical and reproductive categories of deliveries, the optimal caesarean rate was calculated as the median interval (e.g. 51–60%).

The magnitude of variation in optimal rates was examined by stratifying the median optimal caesarean rate according to national caesarean rate and secondary explanatory variables (occupation, geographical region of main experience, country income level, facility type, highest facility level, facility caesarean rate, gender and age). Associations between national caesarean rate and category-specific optimal rates were also examined by calculating the median optimal rate for each category of deliveries stratified by national caesarean rate. Kruskal-Wallis one-way analysis of variance tests were used to investigate differences in opinions of the optimal rate between strata, due to the skewness of responses. All analyses were conducted in Stata version 13.

## Results

### Sample description

A total of 1,377 respondents accessed the link to the survey, of which 320 (23%) questionnaires were excluded as the respondent did not answer any question relating to optimal caesarean rates. The final sample included 1,057 medical doctors from 96 countries (Table 1). The majority of respondents were obstetricians (88%), with an additional 5% other clinical doctors, and 6% researchers not currently involved in clinical practice but who had performed a caesarean in the past five years. One third of respondents (34%) had practiced obstetrics primarily in the Americas; 27% were from Europe and 14% from Africa. The region with the smallest number of respondents was South-East Asia (n = 67, 6%). Most respondents (83%) had mainly practiced in countries with a caesarean rate above 15%, while 7% practiced in countries with national rates below 5%. Half (50%) of respondents practiced in public facilities only, 29% in private facilities only (either for- or not-for-profit), and 19% in both. The highest facility level of practice was national or university hospitals (44%), followed by regional hospitals (27%) and private/other facilities (15%). Forty-two percent estimated that the caesarean rate in their facility was between 15–29% of deliveries, and another 30% estimated it to be between 30–49%.

**Table 1:** Description of respondents to online survey on obstetric care providers’ opinions of the optimal caesarean rate.

Characteristics	Number in final sample (%)
Total	1,057
**Occupation**	
Obstetrician	932 (88.2)
Other clinical doctor	57 (5.4)
Other (including non-clinical doctor and researcher)	60 (5.7)
Missing	8 (0.8)
**Region of main experience in obstetrics**	
Africa	147 (13.9)
Americas	364 (34.4)
Eastern Mediterranean	71 (6.7)
Europe	283 (26.8)
South-East Asia	67 (6.3)
Western Pacific	110 (10.4)
Missing	15 (1.4)
**National caesarean rate in country of practice**	
<5%	75 (7.1)
5–15%	89 (8.4)
15–30%	489 (46.3)
≥30%	385 (36.4)
Missing	19 (1.8)
**Country of practice income level**	
Low income	118 (11.2)
Lower middle income	148 (14.0)
Upper middle income	414 (39.2)
High income	362 (34.2)
Missing	15 (1.4)
**Type of facility of practice**	
Public only	525 (49.7)
Private for-profit only	221 (20.9)
Private not-for-profit only	73 (6.9)
Mixed private	13 (1.2)
Mixed public-private	204 (19.3)
Missing	21 (2.0)
**Highest facility level of practice**	
Primary care	32 (3.0)
District	115 (10.9)
Regional	286 (27.1)
National/University	461 (43.6)
Private/Other	158 (14.9)
Missing	5 (0.5)
**Facility caesarean rate**	
0–14%	89 (8.4)
15–29%	446 (42.2)
30–49%	315 (29.8)
50%+	174 (16.5)
Don’t know	23 (2.2)
Missing	10 (0.9)
**Gender**	
Female	482 (45.6)
Male	560 (53.0)
Missing	15 (1.4)
**Age**	
20–29	52 (4.9)
30–39	262 (24.8)
40–49	306 (28.9)
50–59	289 (27.3)
60+	141 (13.3)
Missing	7 (0.7)
**Language in which completed survey**	
English	657 (62.2)
French	71 (6.7)
Spanish	245 (23.2)
Portuguese	84 (7.9)

### Optimal caesarean rate for all deliveries

Table 2 presents the median reported optimal caesarean rate and IQR, stratified by respondent characteristics. The optimal caesarean rate for all deliveries was missing for 11% of respondents (n = 116), including four respondents who replied that the optimal rate is “less than” a specific percentage (namely 5%, 20%, 20% and 25%) without giving a lower limit, and three who reported that it is “impossible to know” or depends on the population, leaving 941 respondents in the analysis.

**Table 2:** Optimal caesarean rates for all deliveries reported by obstetric care providers, stratified by respondent characteristics (N = 941).

Characteristic	Number of respondents	Median reported optimal caesarean rate (% all deliveries)	Inter-quartile range (IQR, % all deliveries)	Kruskal-Wallis p-value
Total sample	941	20	15–30	-
**Occupation**				0.0002
Obstetrician	835	20	15–30	
Other clinical doctor	47	20	15–40	
Other (including non-clinical doctor and researcher)	53	15	15–22	
**Region of main experience in obstetrics**				<0.0001
Africa	127	20	15–30	
Americas	337	25	20–30	
Eastern Mediterranean	61	23	18–30	
Europe	246	15	14–20	
South-East Asia	64	25	20–30	
Western Pacific	94	25	20–30	
**National caesarean rate in country of practice**				<0.0001
<5%	65	20	15–25	
5–15%	76	20	15–30	
15–30%	434	20	15–30	
> = 30%	350	25	20–30	
**Country of practice income level**				<0.0001
Low income	103	20	15–25	
Lower middle income	131	25	16–30	
Upper middle income	381	28	20–30	
High income	314	17	15–25	
**Type of facility of practice**				<0.0001
Public only	469	20	15–25	
Private for-profit only	187	30	20–35	
Private not-for-profit only	65	20	15–30	
Mixed private	13	25	20–30	
Mixed public-private	189	23	16–30	
**Highest facility level of practice**				<0.0001
Primary care	30	25	18–30	
District	94	20	15–25	
Regional	261	20	15–30	
National/University	413	20	15–30	
Private/Other	139	30	23–35	
**Facility caesarean rate**				<0.0001
0–14%	76	15	10–16	
15–29%	396	20	15–25	
30–49%	285	25	20–30	
50%+	156	30	24–40	
Don’t know	19	20	15–35	
**Gender**				0.0081
Female	429	20	15–30	
Male	502	25	15–30	
**Age**				0.2405
20–29	44	20	15–30	
30–39	237	20	15–30	
40–49	266	20	15–30	
50–59	264	25	15–30	
60+	126	20	15–30	

The median reported optimal caesarean rate for all deliveries was 20% (IQR: 15–30%, range: 3–90%). There was strong evidence of an association between optimal reported rates and all explanatory variables (Kruskal-Wallis p<0.01 for all), except for age. Providers in countries with caesarean rates above 30% reported higher optimal rates than those in countries with caesarean rates below 30% (25%, compared with 20% in all three groups below 30%), while respondents practicing in Europe reported lower optimal rates than elsewhere (15%, compared with at least 20% in other regions). Providers exclusively practicing in the private for-profit sector reported higher optimal rates than those practicing exclusively in the public sector (30% compared with 20%, respectively). Median reported optimal rates increased consistently with reported facility caesarean rates, from 15% among providers who report an institutional caesarean rate of 0–14%, to 30% for institutional rates over 50%.

For each of these stratifications, the 25^th^ percentile in each subgroup was at least 15%; the only exceptions were providers in Europe and in facilities with institutional caesarean rates below 15%, where the lower quartile was 14% and 10%, respectively.

### Optimal caesarean rate among categories of deliveries

**Fig 1:**
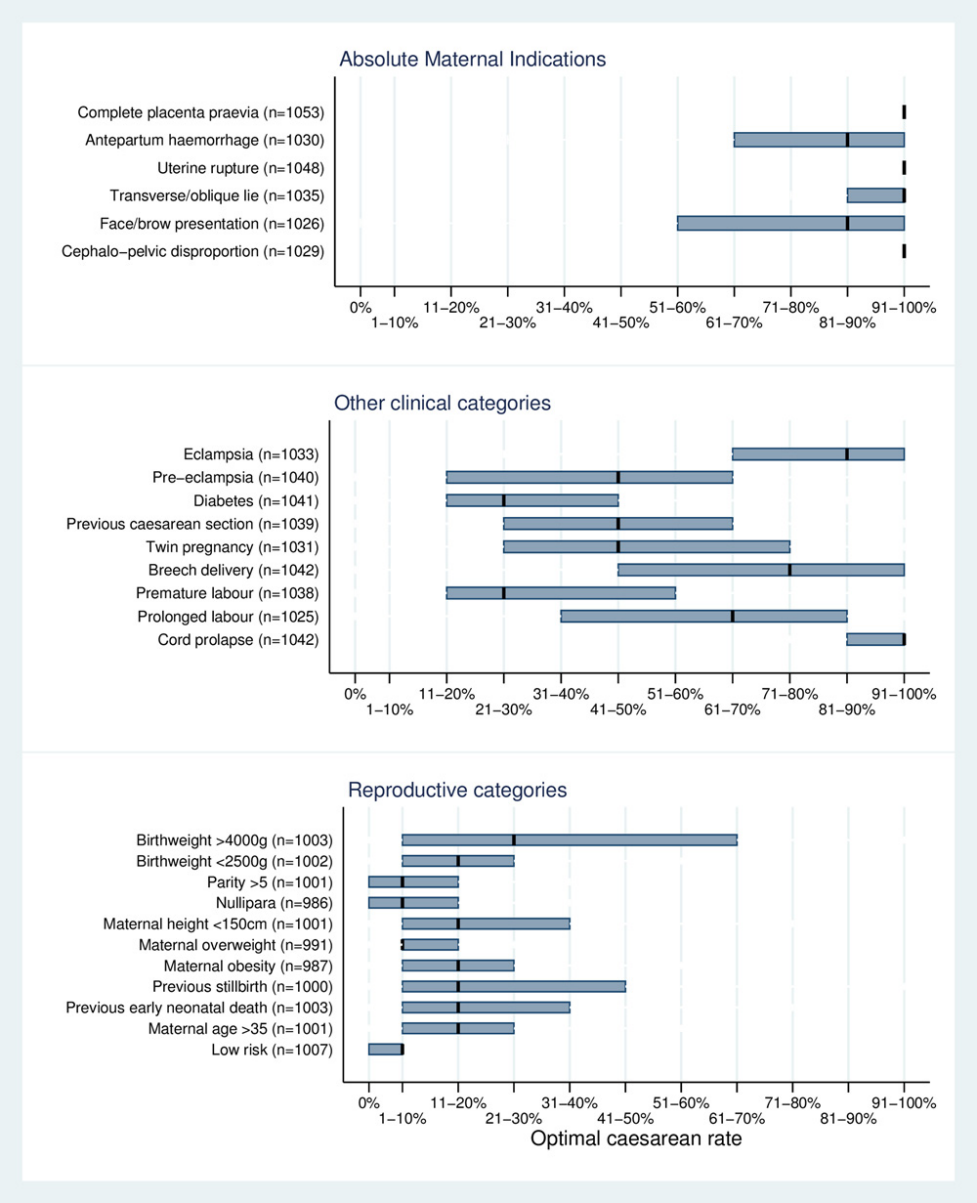
Median optimal caesarean rate and inter-quartile range among different categories of deliveries.

The percentage of missing values for optimal rates ranged from 0.4% for complete placenta praevia to 6.7% for nulliparous women among reproductive categories. Fig 1 presents the median reported optimal caesarean rate and IQR for different categories of deliveries, gathered into three groups (AMIs, other clinical categories, and reproductive categories including low-risk). Four of the six AMIs had median rates of 91–100% (complete placenta praevia, uterine rupture, transverse/oblique lie and cephalopelvic disproportion), while antepartum haemorrhage from placental abruption and face/brow presentation had median optimal rates of 81–90%. There was very little variation in the optimal caesarean rate within these categories, as indicated by the narrow IQRs. Antepartum haemorrhage from placental abruption and face/brow presentation both had a median optimal rate of 81–90%, and wider IQRs than the four other AMI categories.

There was substantial variation in reported optimal rates in most of the other clinical categories. The two exceptions were eclampsia and cord prolapse, with high optimal rates (81–90% and 91–100%, respectively) and relatively narrow IQRs. For the other categories included in this group, the median optimal rate varied between 21–30% for diabetes and premature labour, and 71–80% for breech delivery. The IQRs for these categories were very wide, reaching 50 percentage points for pre-eclampsia, twin delivery, breech delivery and prolonged labour.

The reported optimal rate was lower for reproductive categories than for clinical categories, with medians ranging between 1–10% and 21–30%, and they also tended to show less variation than clinical categories (other than AMIs), with the exception of high birthweight which had an IQR of 60 percentage points. The reported optimal rate was lower for low-risk deliveries than for all other clinical and reproductive categories (p<0.001 for all).

## Discussion

This was the first global survey of obstetricians’ opinions of the optimal caesarean rate. The median optimal caesarean rate reported by obstetricians was 20% (IQR = 15–30%) for all deliveries. The lower quartile of 15% indicates that 75% of respondents consider the optimal caesarean rate at the population level to be higher than the WHO “acceptable” range of 5–15%. There remains substantial variation in opinions of the optimal rate, with one quarter of respondents believing it is above 30%. Factors affecting variation in reported optimal rates include global region of practice, country income level, facility level, and facility caesarean rate. Reported optimal caesarean rates across different categories of deliveries are consistent with clinical interpretation, with very high median rates for AMIs and the lowest reported rates among multipara with singleton, cephalic delivery and no known risk factors.

Although recent evidence suggests that mortality may continue to decline with caesarean rates above the WHO 15% upper threshold [16], in line with the opinions reported in our sample, the findings from this survey cannot be used to infer the “true” optimal caesarean rate, since obstetricians’ opinions of the optimal rate may be biased by their experience of clinical practice. The true optimal caesarean rate—if such a constant optimal rate exists across populations—remains unknown. The variation in reported optimal rates suggests that obstetricians agree on a minimal caesarean rate at the population level (99% of respondents considered the optimal rate to be 7.5% or higher) corresponding to absolute indications, but probably disagree on the risk-benefit balance of caesareans for other indications and on maternal request.

The median rates of 20% and 30% among public and private for-profit providers respectively are consistent with the public-private gap in “ideal” rates of 16% and 20% reported by South African obstetricians in a 1992 survey, although reported optimal rates in both groups were higher in our survey [19]. By definition, the optimal caesarean rate should not vary between public and private sectors; this finding suggests that obstetric care providers’ opinions of the optimal caesarean rate are influenced by the norms and culture surrounding caesarean sections in their facilities. Caesarean rates have been found to be higher in private than public facilities worldwide [37], and a number of factors are likely to drive this trend, including financial incentives, fear of litigation, a desire to enhance patient satisfaction, convenience of scheduling, and staffing patterns in private facilities [27, 38–40]. The high caesarean rates in private facilities may become normalised and shape providers’ perception of the risks associated with caesareans [23, 27]. Accordingly, the only group other than private obstetricians reporting an optimal rate of 30% or higher was providers practicing in facilities where they estimated that at least half of births were by caesarean.

Three groups of respondents reported relatively low optimal caesarean rates (≤15%), including obstetricians no longer practicing medicine (predominantly academic researchers and international non-governmental organisation employees), perhaps because they are more aware of the debates surrounding the risks of excessive caesareans in the academic literature. The second group is obstetricians predominantly practicing in Europe. Of the 246 respondents in this group, the largest contributions came from Norway (n = 95), the UK (n = 47) and Switzerland (n = 25). Few respondents had practiced in Eastern or Southern Europe, where caesarean rates are higher, which may have biased this finding. Western European countries have historically favoured midwife-centred delivery models and public healthcare systems, whereas in the Americas and certain middle-income countries, private insurance systems, obstetrician-led models of care, and higher utilisation of private facilities are thought to incentivise surgical deliveries [39, 41, 42]. Lastly, obstetricians practicing in facilities with low caesarean rates also report optimal rates of 15% regardless of country income level.

The extent of variation in reported rates, even among clinical groups of indications with solid available evidence for planned caesarean delivery, raises questions about the validity of recommendations based on expert opinion, such as those emanating from Delphi consultations [43]. For example, a quarter of respondents believed that fewer than 50% of women with breech delivery should deliver by caesarean, despite a systematic review showing improved perinatal survival and lower severe neonatal morbidity with planned caesarean at term [44]. Though the trials showed these benefits are lower in areas with high perinatal mortality, reported optimal rates for breech delivery still varied substantially among respondents from high-income countries, where perinatal mortality is low (results not shown). Variation in responses in other categories are less surprising: for example, prolonged labour is an imprecise clinical condition with diverse causes and multiple case definitions, while eclampsia can be managed by inducing labour, and therefore variations in responses are likely to reflect to some extent variations in induction practices. The range of responses provided in this survey highlight a need for defining what conditions need to be met for a Delphi consultation to produce valid recommendations, and how to select respondents for the consultation.

A number of limitations to our study are worth noting. First, our sample was not globally representative of doctors performing caesareans. The dissemination strategy achieved a large and geographically diverse sample, though there were relatively few respondents from Asia (in particular, there were only two obstetricians practicing in China in the sample). It was not possible to calculate a response rate for this online survey since the number of people who received the invitation is unknown. Selection bias may have affected the study findings if those who answered the survey tend to report different answers than those who are not reachable online or who chose not to answer the survey. Notably, the lack of responses from southern and eastern Europe may have biased the median reported optimal rate in this region. Furthermore, respondents may have misreported their opinions of optimal caesarean rates through survey fatigue or bias from personal experience (for example, if obstetricians practicing in high-risk referral hospitals overestimated optimal rates), potentially leading to information bias. The survey design attempted to minimise the likelihood of information bias by beginning the questionnaire with the most clear-cut categories (AMIs) and ending with the population-level rate.

Second, the sample was restricted to medical doctors who perform caesareans. Though non-physician clinicians perform a substantial proportion of caesareans in several countries such as Malawi, Mozambique, and Tanzania (up to 93% in some facilities) [45, 46], their training and scope of responsibilities varies substantially from country to country [47]. We restricted the sample to medical doctors in an attempt to maintain a standardised sample across countries. Third, respondents were not asked for their opinion of the WHO guidelines, and it was not possible to assess directly their opinion of the 5–15% recommended range. Lastly, missing values increased with question order due to respondent attrition, and the 11% missing responses for the optimal rate among all deliveries could have been reduced by placing this question before specific delivery categories.

## Conclusions

Overall, the median optimal caesarean rate reported by obstetricians lies above the “acceptable” range defined by the WHO, although the “true” optimal caesarean rate—if indeed one exists across populations—remains unknown. The wide range of reported optimal rates indicates that there is little consensus among obstetric care providers as to the optimal caesarean rate, with the exception of Absolute Maternal Indications (for which caesareans are considered necessary). The recent WHO statement recognised the pitfalls of issuing population-level recommendations for caesarean rates, urging to focus efforts on “provid[ing] caesarean sections to women in need, rather than striving to achieve a specific rate” [14]. Nonetheless, our findings indicate that there is little consensus among obstetricians regarding which women are “in need” of a caesarean. Future work should expand on the validation of approaches aiming to determine the need for caesareans among sub-groups of women, such as the Robson classification.

## Supporting Information

S1 Table Most recent national caesarean section rate estimates at the time of the survey for all countries with available source, updated from Gibbons et al. (DOCX)
