# Supplementary material for: Obstetricians’ Opinions of the Optimal Caesarean Rate: A Global Survey
Source: PLoS One. 2016 Mar 31;11(3):e0152779. doi: 10.1371/journal.pone.0152779 (PMC4816518; doi:10.1371/journal.pone.0152779)
Supplement: S1 Table — (DOCX) [file pone.0152779.s001.docx]

S1 Table: Most recent national caesarean section rate estimates at the time of the survey for all countries with available source, updated from Gibbons et al. [^32^](#_ENREF_32)

| **Country** | **Caesarean rate (%)** | **Year** | **Source** |
| --- | --- | --- | --- |
| Afghanistan | **3.6** | 2011 | World Health Organization. Global Health Observatory data repository. Available at: http://www.who.int/gho/countries/en/. |
| Albania | **18.7** | 2008-2009 | Institute of Statistics, Institute of Public Health [Albania] and ICF Macro. 2010. Albania Demographic and Health Survey 2008-09. Tirana, Albania: Institute of Statistics, Institute of Public Health and ICF Macro. Available at: http://dhsprogram.com/publications/publication-FR230-DHS-Final-Reports.cfm |
| Algeria | **6** | 2000 | World Health Organization. The world health report 2005. Basic indicators. Available at: http://www.who.int/whr/2005/annex/indicators_country_a-f.pdf. |
| Andorra | **23.7** | 1999 | World Health Organization. European Regional Office Health for All database. Available at: http://data.euro.who.int/hfadb. |
| Argentina | **35.2** | 2005 | Villar J, Valladares E, Wojdyla D, et al. Caesarean delivery rates and pregnancy outcomes: the 2005 WHO global survey on maternal and perinatal health in Latin America. Lancet 2006;367:1819-29 |
| Armenia | **12.5** | 2010 | National Statistical Service [Armenia], Ministry of Health [Armenia], and ICF International. 2012. Armenia Demographic and Health Survey 2010. Calverton, Maryland: National Statistical Service, Ministry of Health, and ICF International. Available at: http://dhsprogram.com/publications/publication-FR252-DHS-Final-Reports.cfm |
| Australia | **31.5** | 2009 | World Health Organization. Global Health Observatory data repository. Available at: http://www.who.int/gho/countries/en/. |
| Austria | **29** | 2011 | World Health Organization. Global Health Observatory data repository. Available at: http://www.who.int/gho/countries/en/. |
| Azerbaijan | **7.6** | 2007 | World Health Organization. European Regional Office Health for All database. Available at: http://data.euro.who.int/hfadb. |
| Bahrain | **26.8** | 2010 | World Health Organization. Global Health Observatory data repository. Available at: http://www.who.int/gho/countries/en/. |
| Bangladesh | **17.1** | 2011 | National Institute of Population Research and Training (NIPORT), Mitra and Associates, and ICF International. 2013. Bangladesh Demographic and Health Survey 2011. Dhaka, Bangladesh and Calverton, Maryland, USA: NIPORT, Mitra and Associates, and ICF International. Available at: http://dhsprogram.com/publications/publication-FR265-DHS-Final-Reports.cfm |
| Belarus | **23** | 2010 | World Health Organization. Global Health Observatory data repository. Available at: http://www.who.int/gho/countries/en/. |
| Belgium | **20.3** | 2008 | World Health Organization. Global Health Observatory data repository. Available at: http://www.who.int/gho/countries/en/. |
| Benin | **3.6** | 2006 | Institut National de la Statistique et de l'Analyse Économique (INSAE) [Bénin] et Macro International Inc. 2007: Enquête Démographique et de Santé (EDSB-III) - Bénin 2006. Calverton, MD: Institut National de la Statistique et de l'Analyse Économique et Macro International Inc. Available at: http://www.measuredhs.com/pubs/pdf/FR197/08Chapitre08.pdf. |
| Bhutan | **12.4** | 2010 | World Health Organization. Global Health Observatory data repository. Available at: http://www.who.int/gho/countries/en/. |
| Bolivia | **18.6** | 2008 | Ministerio de Salud y Deportes (MSD), Programa Reforma de Salud (PRS), Instituto Nacional de Estadística (INE) y Macro International. 2009. Encuesta Nacional de Demografía y Salud ENDSA 2008. La Paz, Bolivia: MSD, PRS, INE y Macro International. Available at:http://www.measuredhs.com/pubs/pdf/FR228/FR228%5B08Feb2010%5D.pdf. |
| Bosnia | **18.6** | 2010 | World Health Organization. Global Health Observatory data repository. Available at: http://www.who.int/gho/countries/en/. |
| Brazil | **52.3** | 2010 | World Health Organization. Global Health Observatory data repository. Available at: http://www.who.int/gho/countries/en/. |
| Bulgaria | **31** | 2010 | World Health Organization. Global Health Observatory data repository. Available at: http://www.who.int/gho/countries/en/. |
| Burkina Faso | **1.81** | 2010 | Institut National de la Statistique et de la Démographie (INSD) et ICF International, 2012. Enquête Démographique et de Santé et à Indicateurs Multiples du Burkina Faso 2010. Calverton, Maryland, USA : INSD et ICF International. Available at: http://dhsprogram.com/publications/publication-FR256-DHS-Final-Reports.cfm |
| Burundi | **4** | 2010 | Institut de Statistiques et d’Études Économiques du Burundi (ISTEEBU), Ministère de la Santé Publique et de la Lutte contre le Sida [Burundi] (MSPLS), et ICF International. 2012. Enquête Démographique et de Santé Burundi 2010. Bujumbura, Burundi : ISTEEBU, MSPLS, et ICF International. Available from: http://dhsprogram.com/publications/publication-FR253-DHS-Final-Reports.cfm |
| Cambodia | **3** | 2010 | National Institute of Statistics, Directorate General for Health, and ICF Macro, 2011. Cambodia Demographic and Health Survey 2010. Phnom Penh, Cambodia and Calverton, Maryland, USA: National Institute of Statistics, Directorate General for Health, and ICF Macro. Available from: http://dhsprogram.com/publications/publication-FR249-DHS-Final-Reports.cfm |
| Cameroon | **3.8** | 2011 | Institut National de la Statistique (INS) et ICF. International. 2012. Enquête Démographique et de Santé et à Indicateurs Multiples du Cameroun 2011. Calverton, Maryland, USA : INS et ICF International. Available at: http://dhsprogram.com/publications/publication-FR260-DHS-Final-Reports.cfm |
| Canada | **27.8** | 2010 | World Health Organization. Global Health Observatory data repository. Available at: http://www.who.int/gho/countries/en/. |
| Cape Verde | **10.7** | 2005 | Instituto Nacional de Estatística (INE) [Cabo Verde], Ministério da Saúde, e Macro International 2008. Segundo Inquérito Demográfico e de Saúde Reprodutiva, Cabo Verde, IDSR-II, 2005. Calverton, MD: INE Available at:http://www.measuredhs.com/pubs/pdf/FR203/FR203.pdf. |
| Central African Republic | **1.9** | 1994-1995 | Mboup G. 1995. Enquête Démographique et de Santé, République Centrafrieaine 1994-95. Calverton, MD: Direction des Statistiques Démographiques et Sociales et Macro International Inc. Available at:http://www.measuredhs.com/pubs/pdf/FR67/10Chapitre10.pdf. |
| Chad | **1.5** | 2010 | World Health Organization. Global Health Observatory data repository. Available at: http://www.who.int/gho/countries/en/. |
| Chile | **37** | 2010 | World Health Organization. Global Health Observatory data repository. Available at: http://www.who.int/gho/countries/en/. |
| China | **27** | 2008 | World Health Organization. Global Health Observatory data repository. Available at: http://www.who.int/gho/countries/en/. |
| Colombia | **42.8** | 2011 | World Health Organization. Global Health Observatory data repository. Available at: http://www.who.int/gho/countries/en/. |
| Comoros | **5.3** | 1996 | Mondoha, Kassim A, Schoemaker J, Ban'ère M. 1997. Enquête Démographique et de Santé, Comores 1996. Calverton, MD: Centre National de Documentation et de Recherche Scientifique et Macro International Inc. Available at: http://www.measuredhs.com/pubs/pdf/FR79/07Chapitre7.pdf. |
| Congo Democratic Republic | **7.2** | 2010 | World Health Organization. Global Health Observatory data repository. Available at: http://www.who.int/gho/countries/en/. |
| Congo (Republic) | **3.2** | 2005 | Centre National de la Statistique et des Études Économiques (CNSEE) et ORC Macro. 2006. Enquête Démographique et de Santé du Congo 2005. Calverton, Maryland, USA : CNSEE et ORC Macro. Available at: http://dhsprogram.com/publications/publication-FR182-DHS-Final-Reports.cfm |
| Costa Rica | **20.9** | 2010 | World Health Organization. Global Health Observatory data repository. Available at: http://www.who.int/gho/countries/en/. |
| Cote d′Ivoire | **4.6** | 2005 | Institut National de la Statistique (INS) et Ministère de la Lutte contre le Sida [Côte d'Ivoire] et ORC Macro. 2006. Enquête sur les Indicateurs du Sida, Côte d'Ivoire 2005. Calverton, MD: INS et ORC Macro. Available at:http://www.measuredhs.com/pubs/pdf/AIS5/AIS5.pdf. |
| Croatia | **18.7** | 2010 | World Health Organization. Global Health Observatory data repository. Available at: http://www.who.int/gho/countries/en/. |
| Cuba | **35.6** | 2005 | Villar J, Valladares E, Wojdyla D, et al. Caesarean delivery rates and pregnancy outcomes: the 2005 WHO global survey on maternal and perinatal health in Latin America. Lancet 2006;367:1819-29 |
| Cyprus | **50.9** | 2007 | World Health Organization. Global Health Observatory data repository. Available at: http://www.who.int/gho/countries/en/. |
| Czech Republic | **24.1** | 2010 | World Health Organization. Global Health Observatory data repository. Available at: http://www.who.int/gho/countries/en/. |
| Denmark | **21** | 2011 | World Health Organization. Global Health Observatory data repository. Available at: http://www.who.int/gho/countries/en/. |
| Dominican Republic | **38.8** | 2010 | World Health Organization. Global Health Observatory data repository. Available at: http://www.who.int/gho/countries/en/. |
| [Ecuadorb](http://www.sciencedirect.com/science/article/pii/S000293781200258X#tblfn2) | **29.8** | 2005 | Villar J, Valladares E, Wojdyla D, et al. Caesarean delivery rates and pregnancy outcomes: the 2005 WHO global survey on maternal and perinatal health in Latin America. Lancet 2006;367:1819-29. |
| Egypt | **27.6** | 2008 | El-Zanaty F, Way A. 2009. Egypt Demographic and Health Survey 2008. Cairo, Egypt: Ministry of Health, El-Zanaty and Associates, and Macro International (. Available at:http://www.measuredhs.com/pubs/pdf/FR220/FR220.pdf) |
| El Salvador | **25** | 2008 | Asociación Demográfica Salvadoreña, CDC, USAID. República de El Salvador, CA. Encuesta Nacional de Salud Familiar. Informe final. FESAL-2008 |
| Eritrea | **2.7** | 2002 | National Statistics and Evaluation Office (NSEO) [Eritrea] and ORC Macro. 2003. Eritrea Demographic and Health Survey 2002. Calverton, MD: National Statistics and Evaluation Office and ORC Macro. Available at:http://www.measuredhs.com/pubs/pdf/FR137/09Chapter09.pdf. |
| Estonia | **20.2** | 2011 | World Health Organization. Global Health Observatory data repository. Available at: http://www.who.int/gho/countries/en/. |
| Ethiopia | **1.4** | 2011 | Central Statistical Agency [Ethiopia] and ICF International. 2012. Ethiopia Demographic and Health Survey 2011. Addis Ababa, Ethiopia and Calverton, Maryland, USA: Central Statistical Agency and ICF International. Available at: http://dhsprogram.com/publications/publication-FR255-DHS-Final-Reports.cfm |
| Finland | **16.3** | 2011 | World Health Organization. Global Health Observatory data repository. Available at: http://www.who.int/gho/countries/en/. |
| France | **21** | 2010 | World Health Organization. Global Health Observatory data repository. Available at: http://www.who.int/gho/countries/en/. |
| Gabon | **10** | 2012 | Direction Générale de la Statistique (DGS) et ICF International. 2013. Enquête émographique et de Santé du Gabon 2012. Calverton, Maryland, et Libreville, Gabon : DGS et ICF International. Available at: http://dhsprogram.com/publications/publication-FR276-DHS-Final-Reports.cfm |
| Gambia | **2.5** | 2010 | World Health Organization. Global Health Observatory data repository. Available at: http://www.who.int/gho/countries/en/. |
| Georgia | **23.9** | 2010 | World Health Organization. Global Health Observatory data repository. Available at: http://www.who.int/gho/countries/en/. |
| Germany | **32.1** | 2011 | World Health Organization. Global Health Observatory data repository. Available at: http://www.who.int/gho/countries/en/. |
| Ghana | **6.9** | 2008 | Ghana Statistical Service (GSS), Ghana Health Service (GHS), and ICF Macro. 2009. Ghana Demographic and Health Survey 2008. Accra, Ghana: GSS, GHS, and ICF Macro. Available at:http://www.measuredhs.com/pubs/pdf/FR221/FR221.pdf. |
| Guatemala | **16.3** | 2009 | World Health Organization. Global Health Observatory data repository. Available at: http://www.who.int/gho/countries/en/. |
| Guinea | **2.4** | 2007 | World Health Organization. Global Health Observatory data repository. Available at: http://www.who.int/gho/countries/en/. |
| Guyana | **13.3** | 2009 | Ministry of Health (MOH), Bureau of Statistics (BOS), and ICF Macro. 2010. Guyana Demographic and Health Survey 2009. Georgetown, Guyana: MOH, BOS, and ICF Macro. Available at: http://dhsprogram.com/publications/publication-FR232-DHS-Final-Reports.cfm |
| Haiti | **5.5** | 2012 | Cayemittes, Michel, Michelle Fatuma Busangu, Jean de Dieu Bizimana, Bernard Barrère, Blaise Sévère, Viviane Cayemittes et Emmanuel Charles. 2013. Enquête Mortalité, Morbidité et Utilisation des Services, Haïti, 2012. Calverton, Maryland, USA : MSPP, IHE et ICF International. Available at: http://dhsprogram.com/publications/publication-FR273-DHS-Final-Reports.cfm |
| Honduras | **18.6** | 2011-12 | Secretaría de Salud [Honduras], Instituto Nacional de Estadística (INE) e ICF International. 2013. Encuesta Nacional de Salud y Demografía 2011-2012. Tegucigalpa, Honduras: SS, INE e ICF International. Available at: http://dhsprogram.com/publications/publication-FR274-DHS-Final-Reports.cfm |
| Hungary | **28** | 2007 | World Health Organization. European Regional Office Health for All database. Available at: http://data.euro.who.int/hfadb. |
| Iceland | **16.6** | 2011 | World Health Organization. Global Health Observatory data repository. Available at: http://www.who.int/gho/countries/en/. |
| India | **8.1** | 2008 | World Health Organization. Global Health Observatory data repository. Available at: http://www.who.int/gho/countries/en/. |
| Indonesia | **6.8** | 2007 | Statistics Indonesia (Badan Pusat Statistik-BPS) and Macro International. 2008. Indonesia Demographic and Health Survey 2007. Calverton, MD: BPS and Macro International. Available at:http://www.measuredhs.com/pubs/pdf/FR218/FR218%5BApril-09-2009%5D.pdf. |
| Iran | **40** | 2005 | World Health Organization. Global Health Observatory data repository. Available at: http://www.who.int/gho/countries/en/. |
| Ireland | **26.3** | 2010 | World Health Organization. Global Health Observatory data repository. Available at: http://www.who.int/gho/countries/en/. |
| Israel | **19.9** | 2010 | World Health Organization. Global Health Observatory data repository. Available at: http://www.who.int/gho/countries/en/. |
| Italy | **38** | 2009 | World Health Organization. Global Health Observatory data repository. Available at: http://www.who.int/gho/countries/en/. |
| Jamaica | **14.8** | 2009 | World Health Organization. Global Health Observatory data repository. Available at: http://www.who.int/gho/countries/en/. |
| Japan | **23.3** | 2008 | World Health Organization. Global Health Observatory data repository. Available at: http://www.who.int/gho/countries/en/. |
| Jordan | **18.5** | 2007 | Department of Statistics [Jordan] and Macro International Inc. 2008. Jordan Population and Family Health Survey 2007. Calverton, MD: Department of Statistics and Macro International Inc. Available at:http://www.measuredhs.com/pubs/pdf/FR209/FR209.pdf. |
| Kazakhstan | **13.5** | 2010 | World Health Organization. Global Health Observatory data repository. Available at: http://www.who.int/gho/countries/en/. |
| Kenya | **5.81** | 2009 | Kenya National Bureau of Statistics (KNBS) and ICF Macro. 2010. Kenya Demographic and Health Survey 2008-09. Calverton, Maryland: KNBS and ICF Macro. Available at: http://dhsprogram.com/publications/publication-FR229-DHS-Final-Reports.cfm |
| Kuwait | **11.2** | 1996 | Alnesef Y, Al-Rashoud RH, Farid SM. Kuwait Family Health Survey 1996. Kuwait: Ministry D71 of Health; 2000. |
| Kyrgyzstan | **6.9** | 2010 | World Health Organization. Global Health Observatory data repository. Available at: http://www.who.int/gho/countries/en/. |
| Laos | **3.7** | 2011-12 | Department of Statistics and UNICEF. 2012. Lao PDR Multiple Indicator Cluster Survey 2011-12, Final Report. Vientiane, Lao PDR: Department of Statistics and UNICEF. Available at: http://dhsprogram.com/publications/publication-FR268-Other-Final-Reports.cfm |
| Latvia | **23.9** | 2010 | World Health Organization. Global Health Observatory data repository. Available at: http://www.who.int/gho/countries/en/. |
| Lebanon | **23.3** | 2000 | Betrán AP, Merialdi M, Lauer JA, et al. Rates of caesarean section: analysis of global, regional and national estimates. Paediatr Perinat Epidemiol 2007;21:98-113 |
| Lesotho | **6.5** | 2009 | Ministry of Health and Social Welfare (MOHSW) [Lesotho] and ICF Macro. 2010. Lesotho Demographic and Health Survey 2009. Maseru, Lesotho: MOHSW and ICF Macro. Available at: http://dhsprogram.com/publications/publication-FR241-DHS-Final-Reports.cfm |
| Liberia | **3.5** | 2007 | Liberia Institute of Statistics and Geo-Information Services (LISGIS) [Liberia], Ministry of Health and Social Welfare [Liberia], National AIDS Control Program [Liberia], and Macro International Inc. 2008. Liberia Demographic and Health Survey 2007. Monrovia, Liberia: Liberia Institute of Statistics and Geo-Information Services (LISGIS) and Macro International Inc. Available at:http://www.measuredhs.com/pubs/pdf/FR201/FR201.pdf. |
| Libyan Arab Jamahiriya | **7.5** | 1995 | Betrán AP, Merialdi M, Lauer JA, et al. Rates of caesarean section: analysis of global, regional and national estimates. Paediatr Perinat Epidemiol 2007;21:98-113 |
| Lithuania | **25.2** | 2010 | World Health Organization. Global Health Observatory data repository. Available at: http://www.who.int/gho/countries/en/. |
| Luxemburg | **31.3** | 2009 | World Health Organization. Global Health Observatory data repository. Available at: http://www.who.int/gho/countries/en/. |
| Madagascar | **1.5** | 2008-09 | Institut National de la Statistique (INSTAT) et ICF Macro. 2010. Enquête Démographique et de Santé de Madagascar 2008-2009. Antananarivo, Madagascar : INSTAT et ICF Macro. Available at: http://dhsprogram.com/publications/publication-FR236-DHS-Final-Reports.cfm |
| Malawi | **4.5** | 2010 | National Statistical Office (NSO) and ICF Macro. 2011. Malawi Demographic and Health Survey 2010. Zomba, Malawi, and Calverton, Maryland, USA: NSO and ICF Macro. Available at: http://dhsprogram.com/publications/publication-FR247-DHS-Final-Reports.cfm |
| Malaysia | **15.7** | 2006 | World Health Organization. Global Health Observatory data repository. Available at: http://www.who.int/gho/countries/en/. |
| Maldives | **32.4** | 2009 | Ministry of Health and Family (MOHF) [Maldives] and ICF Macro. 2010. Maldives Demographic and Health Survey 2009. Calverton, Maryland: MOHF and ICF Macro. Available at: http://dhsprogram.com/publications/publication-FR237-DHS-Final-Reports.cfm |
| Mali | **0.9** | 2006 | Cellule de Planification et de Statistique du Ministère de la Santé (CPS/MS), Direction Nationale de la Statistique et de l'Informatique du Ministère de l'Économie, de l'Industrie et du Commerce (DNSI/MEIC) et Macro International Inc. 2007. Enquête Démographique et de Santé du Mali 2006. Calverton, MD: CPS/DNSI et Macro International Inc. Available at:http://www.measuredhs.com/pubs/pdf/FR199/FR199.pdf. |
| Malta | **31.7** | 2010 | World Health Organization. Global Health Observatory data repository. Available at: http://www.who.int/gho/countries/en/. |
| Marshall Islands | **9.3** | 2007 | World Health Organization. Global Health Observatory data repository. Available at: http://www.who.int/gho/countries/en/. |
| Mauritius | **44.1** | 2010 | World Health Organization. Global Health Observatory data repository. Available at: http://www.who.int/gho/countries/en/. |
| Mauritania | **3.2** | 2000-2001 | Office National de la Statistique (ONS) [Mauritanie] et ORC Macro. 2001. Enquête Démographique et de Santé Mauritanie 2000-2001. Calverton, MD: ONS et ORC Macro. Available at:http://www.measuredhs.com/pubs/pdf/FR127/07Chapter7.pdf. |
| Mexico | **38.8** | 2010 | World Health Organization. Global Health Observatory data repository. Available at: http://www.who.int/gho/countries/en/. |
| Moldova | **14.8** | 2011 | World Health Organization. Global Health Observatory data repository. Available at: http://www.who.int/gho/countries/en/. |
| Mongolia | **21** | 2010 | World Health Organization. Global Health Observatory data repository. Available at: http://www.who.int/gho/countries/en/. |
| Montenegro | **19.3** | 2009 | World Health Organization. Global Health Observatory data repository. Available at: http://www.who.int/gho/countries/en/. |
| Morocco | **16** | 2011 | World Health Organization. Global Health Observatory data repository. Available at: http://www.who.int/gho/countries/en/. |
| Mozambique | **3.9** | 2011 | Ministerio da Saude (MISAU), Instituto Nacional de Estatística (INE) e ICF International (ICFI). Moçambique Inquérito Demográfico e de Saúde 2011. Calverton, Maryland, USA: MISAU, INE e ICFI. Available at: http://dhsprogram.com/publications/publication-FR266-DHS-Final-Reports.cfm |
| Namibia | **12.7** | 2006-2007 | Ministry of Health and Social Services (MoHSS) [Namibia] and Macro International Inc. 2008. Namibia Demographic and Health Survey 2006-07. Windhoek, Namibia and Calverton, MD: MoHSS and Macro International Inc. Available at: http://www.measuredhs.com/pubs/pdf/FR204/FR204.pdf. |
| Nepal | **4.59** | 2011 | Ministry of Health and Population (MOHP) [Nepal], New ERA, and ICF International Inc. 2012. Nepal Demographic and Health Survey 2011. Kathmandu, Nepal: Ministry of Health and Population, New ERA, and ICF International, Calverton, Maryland. Available at: http://dhsprogram.com/publications/publication-FR257-DHS-Final-Reports.cfm |
| Netherlands | **15.4** | 2008 | World Health Organization. Global Health Observatory data repository. Available at: http://www.who.int/gho/countries/en/. |
| New Zealand | **23.6** | 2010 | World Health Organization. Global Health Observatory data repository. Available at: http://www.who.int/gho/countries/en/. |
| [Nicaraguad](http://www.sciencedirect.com/science/article/pii/S000293781200258X#tblfn4) | **19.6** | 2007 | World Health Organization. Global Health Observatory data repository. Available at: http://www.who.int/gho/countries/en/. |
| Niger | **1** | 2006 | Institut National de la Statistique (INS) et Macro International Inc. 2007. Enquête Démographique et de Santé et à Indicateurs Multiples du Niger 2006. Calverton, MD: INS et Macro International Inc. Available at: http://www.measuredhs.com/pubs/pdf/FR193/08Chapitre08.pdf. |
| Nigeria | **1.8** | 2008 | National Population Commission (NPC) [Nigeria] and ICF Macro. 2009. Nigeria Demographic and Health Survey 2008. Abuja, Nigeria: National Population Commission and ICF Macro. Available at:http://www.measuredhs.com/pubs/pdf/FR222/FR222.pdf. |
| Norway | **17.1** | 2010 | World Health Organization. Global Health Observatory data repository. Available at: http://www.who.int/gho/countries/en/. |
| Oman | **16.4** | 2010 | World Health Organization. Global Health Observatory data repository. Available at: http://www.who.int/gho/countries/en/. |
| Pakistan | **7.3** | 2006-2007 | National Institute of Population Studies (NIPS) [Pakistan], and Macro International Inc. 2008. Pakistan Demographic and Health Survey 2006-07. Islamabad, Pakistan: National Institute of Population Studies and Macro International Inc. Available at:http://www.measuredhs.com/pubs/pdf/FR200/FR200.pdf. |
| Panama | **20.2** | 2009 | World Health Organization. Global Health Observatory data repository. Available at: http://www.who.int/gho/countries/en/. |
| [Paraguaya](http://www.sciencedirect.com/science/article/pii/S000293781200258X#tblfn1) | **33.1** | 2008 | World Health Organization. Global Health Observatory data repository. Available at: http://www.who.int/gho/countries/en/. |
| [Peruc](http://www.sciencedirect.com/science/article/pii/S000293781200258X#tblfn3) | **25.3** | 2012 | Instituto Nacional de Estadística e Informática [Peru], and ICF Macro 2012. Encuesta Demográfica y de Salud Familiar 2012. Lima, Peru: Instituto Nacional de Estadística e Informática [Peru], and ICF Macro. Available at: http://dhsprogram.com/publications/publication-FR284-DHS-Final-Reports.cfm |
| Philippines | **9.5** | 2008 | National Statistics Office (NSO) [Philippines], and ICF Macro. 2009. National Demographic and Health Survey 2008. Calverton, MD: National Statistics Office and ICF Macro. Available at:http://www.measuredhs.com/pubs/pdf/FR224/FR224.pdf. |
| Poland | **33.7** | 2010 | World Health Organization. Global Health Observatory data repository. Available at: http://www.who.int/gho/countries/en/. |
| Portugal | **35.8** | 2010 | World Health Organization. Global Health Observatory data repository. Available at: http://www.who.int/gho/countries/en/. |
| Qatar | **15.9** | 1998 | Betrán AP, Merialdi M, Lauer JA, et al. Rates of caesarean section: analysis of global, regional and national estimates. Paediatr Perinat Epidemiol 2007;21:98-113 |
| Republic of Korea | **36.9** | 2009 | World Health Organization. Global Health Observatory data repository. Available at: http://www.who.int/gho/countries/en/. |
| Republic of Moldova | **14.8** | 2011 | World Health Organization. Global Health Observatory data repository. Available at: http://www.who.int/gho/countries/en/. |
| Romania | **30.4** | 2010 | World Health Organization. Global Health Observatory data repository. Available at: http://www.who.int/gho/countries/en/. |
| Russian Federation | **18** | 2006 | World Health Organization. European Regional Office Health for All database. Available at: http://data.euro.who.int/hfadb. |
| Rwanda | **6.94** | 2010 | National Institute of Statistics of Rwanda (NISR) [Rwanda], Ministry of Health (MOH) [Rwanda], and ICF International. 2012. Rwanda Demographic and Health Survey 2010. Calverton, Maryland, USA: NISR, MOH, and ICF International. Available at: http://dhsprogram.com/publications/publication-FR259-DHS-Final-Reports.cfm |
| Samoa | **12.8** | 2009 | Ministry of Health [Samoa], Bureau of Statistics [Samoa], and ICF Macro. 2010. Samoa Demographic and Health Survey 2009. Apia, Samoa: Ministry of Health, Samoa. Available at: http://dhsprogram.com/publications/publication-FR240-DHS-Final-Reports.cfm |
| Sao Tome | **5.3** | 2008-09 | Instituto Nacional de Estatística (INE) [São Tomé e Príncipe], Ministério da Saúde, e ICF Macro. 2010. Inquérito Demográfico e Sanitário, São Tomé e Príncipe, IDS STP, 2008-2009. Calverton, Maryland, USA: INE. Available at: http://dhsprogram.com/publications/publication-FR233-DHS-Final-Reports.cfm |
| Saudi Arabia | **22.3** | 2010 | World Health Organization. Global Health Observatory data repository. Available at: http://www.who.int/gho/countries/en/. |
| Senegal | **4.75** | 2011 | Agence Nationale de la Statistique et de la Démographie (ANSD) [Sénégal], et ICF International. 2012. Enquête Démographique et de Santé à Indicateurs Multiples au Sénégal (EDS-MICS) 2010-2011. Calverton, Maryland, USA: ANSD et ICF International. Available at: http://dhsprogram.com/publications/publication-FR258-DHS-Final-Reports.cfm |
| Serbia | **24.6** | 2010 | World Health Organization. Global Health Observatory data repository. Available at: http://www.who.int/gho/countries/en/. |
| Sierra Leone | **4.5** | 2010 | World Health Organization. Global Health Observatory data repository. Available at: http://www.who.int/gho/countries/en/. |
| Singapore | **30.5** | 2001-2003 | Ministry of Health [Singapore] http://www.nuhgynae.com.sg/cos/o.x?c=/wbn/pagetree&func=view&rid=1073529 |
| Slovakia | **28.7** | 2010 | World Health Organization. Global Health Observatory data repository. Available at: http://www.who.int/gho/countries/en/. |
| Slovenia | **18.2** | 2010 | World Health Organization. Global Health Observatory data repository. Available at: http://www.who.int/gho/countries/en/. |
| Solomon Islands | **6.2** | 2007 | World Health Organization. Global Health Observatory data repository. Available at: http://www.who.int/gho/countries/en/. |
| South Africa | **20.6** | 2003 | Department of Health, Medical Research Council, OrcMacro. 2007. South Africa Demographic and Health Survey 2003. Pretoria: Department of Health. Available at: http://www.measuredhs.com/pubs/pdf/FR206/FR206.pdf. |
| Spain | **25.3** | 2010 | World Health Organization. Global Health Observatory data repository. Available at: http://www.who.int/gho/countries/en/. |
| Sri Lanka | **23.8** | 2007 | World Health Organization. Global Health Observatory data repository. Available at: http://www.who.int/gho/countries/en/. |
| Sudan | **3.7** | 1993 | Betrán AP, Merialdi M, Lauer JA, et al. Rates of caesarean section: analysis of global, regional and national estimates. Paediatr Perinat Epidemiol 2007;21:98-113 |
| Swaziland | **12.3** | 2010 | World Health Organization. Global Health Observatory data repository. Available at: http://www.who.int/gho/countries/en/. |
| Sweden | **16.9** | 2010 | World Health Organization. Global Health Observatory data repository. Available at: http://www.who.int/gho/countries/en/. |
| Switzerland | **32.8** | 2010 | World Health Organization. Global Health Observatory data repository. Available at: http://www.who.int/gho/countries/en/. |
| Syrian Arab Republic | **15** | 2002 | Khawaja M, Choueiry N, Jurdi R. “Hospital-based caesarean section in the Arab region: an overview.” East Mediterr Health J 2009;15:458-69. Available at: http://www.emro.who.int/emhj/1502/15_2_2009_0458_0469.pdf. |
| Taiwan | **33** | 2001 | Liu, T.-C., Chen, C.-S., Tsai, Y.-W. and Lin, H.-C. (2007), Taiwan’s High Rate of Cesarean Births: Impacts of National Health Insurance and Fetal Gender Preference. Birth, 34: 115–122. |
| Tajikistan | **3.4** | 2009 | World Health Organization. Global Health Observatory data repository. Available at: http://www.who.int/gho/countries/en/. |
| Thailand | **17.4** | 2001 | Betrán AP, Merialdi M, Lauer JA, et al. Rates of caesarean section: analysis of global, regional and national estimates. Paediatr Perinat Epidemiol 2007;21:98-113 |
| The FYR of Macedonia | **22.2** | 2010 | World Health Organization. Global Health Observatory data repository. Available at: http://www.who.int/gho/countries/en/. |
| Timor-Leste | **1.7** | 2009-10 | National Statistics Directorate (NSD) [Timor-Leste], Ministry of Finance [Timor-Leste], and ICF Macro 2010. Timor-Leste Demographic and Health Survey 2009-10. Dili, Timor-Leste: NSD [Timor-Leste] and ICF Macro. Available at: http://dhsprogram.com/publications/publication-FR235-DHS-Final-Reports.cfm |
| Togo | **8.8** | 2010 | World Health Organization. Global Health Observatory data repository. Available at: http://www.who.int/gho/countries/en/. |
| Tonga | **11.1** | 2005 | World Health Organization. Global Health Observatory data repository. Available at: http://www.who.int/gho/countries/en/. |
| Tunisia | **20.5** | 2006 | World Health Organization. Global Health Observatory data repository. Available at: http://www.who.int/gho/countries/en/. |
| Turkey | **36.7** | 2008 | World Health Organization. Global Health Observatory data repository. Available at: http://www.who.int/gho/countries/en/. |
| Turkmenistan | **3.8** | 2007 | World Health Organization. Global Health Observatory data repository. Available at: http://www.who.int/gho/countries/en/. |
| Uganda | **5.22** | 2011 | Uganda Bureau of Statistics (UBOS) and ICF International Inc. 2012. Uganda Demographic and Health Survey 2011. Kampala, Uganda: UBOS and Calverton, Maryland: ICF International Inc. Available at: http://dhsprogram.com/publications/publication-FR264-DHS-Final-Reports.cfm |
| Ukraine | **10.4** | 2007 | World Health Organization. Global Health Observatory data repository. Available at: http://www.who.int/gho/countries/en/. |
| United Arab Emirates | **20.9** | 2007 | World Health Organization. Global Health Observatory data repository. Available at: http://www.who.int/gho/countries/en/. |
| United Kingdom | **22** | 2004 | World Health Organization. European Regional Office Health for All database. Available at: http://data.euro.who.int/hfadb. |
| United Republic of Tanzania | **4.25** | 2010 | National Bureau of Statistics (NBS) [Tanzania] and ICF Macro. 2011. Tanzania Demographic and Health Survey 2010. Dar es Salaam, Tanzania: NBS and ICF Macro. Available at: http://dhsprogram.com/publications/publication-FR243-DHS-Final-Reports.cfm |
| United States | **32.8** | 2011 | World Health Organization. Global Health Observatory data repository. Available at: http://www.who.int/gho/countries/en/. |
| Uruguay | **33** | 2007 | World Health Organization. Global Health Observatory data repository. Available at: http://www.who.int/gho/countries/en/. |
| Uzbekistan | **8.6** | 2010 | World Health Organization. Global Health Observatory data repository. Available at: http://www.who.int/gho/countries/en/. |
| Venezuela | **25.1** | 2002 | Betrán AP, Merialdi M, Lauer JA, et al. Rates of caesarean section: analysis of global, regional and national estimates. Paediatr Perinat Epidemiol 2007;21:98-113 |
| Viet Nam | **20** | 2011 | World Health Organization. Global Health Observatory data repository. Available at: http://www.who.int/gho/countries/en/. |
| Yemen | **6.5** | 2009 | World Health Organization. Global Health Observatory data repository. Available at: http://www.who.int/gho/countries/en/. |
| Zambia | **3** | 2007 | Central Statistical Office (CSO), Ministry of Health (MOH), Tropical Diseases Research Centre (TDRC), University of Zambia, and Macro International Inc. 2009. Zambia Demographic and Health Survey 2007. Calverton, MD: CSO and Macro International Inc. Available at:http://www.measuredhs.com/pubs/pdf/FR211/FR211%5Brevised-05-12-2009%5D.pdf). |
| Zimbabwe | **4.44** | 2010-11 | Zimbabwe National Statistics Agency (ZIMSTAT) and ICF International. 2012. Zimbabwe  Demographic and Health Survey 2010-11. Calverton, Maryland: ZIMSTAT and ICF International Inc. Available at: http://dhsprogram.com/publications/publication-FR254-DHS-Final-Reports.cfm |
